# Subjective versus objective risk in genetic counseling for hereditary breast and/or ovarian cancers

**DOI:** 10.1186/1756-9966-28-157

**Published:** 2009-12-21

**Authors:** Anita Caruso, Cristina Vigna, Bruna Marozzo, Fabio M Sega, Isabella Sperduti, Francesco Cognetti, Antonella Savarese

**Affiliations:** 1 Prevention and Training Activities in Psycho-Oncology, National Cancer Institute "Regina Elena", Rome, Italy; 2 Department of Surgery, National Cancer Institute "Regina Elena", Rome, Italy; 3 The Bio-statistical Unit, National Cancer Institute "Regina Elena", Rome, Italy; 4 Department of Medical Oncology, National Cancer Institute "Regina Elena", Rome, Italy

## Abstract

**Background:**

Despite the fact that genetic counseling in oncology provides information regarding objective risks, it can be found a contrast between the subjective and objective risk.

The aims of this study were to evaluate the accuracy of the perceived risk compared to the objective risk estimated by the BRCApro computer model and to evaluate any associations between medical, demographic and psychological variables and the accuracy of risk perception.

**Methods:**

130 subjects were given medical-demographic file, Cancer and Genetic Risk Perception, Hospital Anxiety-Depression Scale. It was also computed an objective evaluation of the risk by the BRCApro model.

**Results:**

The subjective risk was significantly higher than objective risk. The risk of tumour was overestimated by 56%, and the genetic risk by 67%. The subjects with less cancer affected relatives significantly overestimated their risk of being mutation carriers and made a more innacurate estimation than high risk subjects.

**Conclusion:**

The description of this sample shows: general overestimation of the risk, inaccurate perception compared to BRCApro calculation and a more accurate estimation in those subjects with more cancer affected relatives (high risk subjects). No correlation was found between the levels of perception of risk and anxiety and depression. Based on our findings, it is worth pursuing improved communication strategies about the actual cancer and genetic risk, especially for subjects at "intermediate and slightly increased risk" of developing an hereditary breast and/or ovarian cancer or of being mutation carrier.

## Background

Oncological genetic counseling enables to discover a hereditary component which increases the risk of developing a tumour. The concept of risk is particularly important in this process.

The probability that an event occurs can be estimated subjectively through the perception that a single individual has of the risk. Alternatively, it can be measured objectively using well-defined parameters.

In oncological genetic counseling, two reasons make it important to measure the objective risk of having a genetic mutation which increases the risk of developing a tumour: it makes it possible to carry out a mutation analysis only on eligible people and also creates suitable prevention programmes for different levels of risk.

Subjective risk assessment has also a great importance because it influences decisions on whether to undergo genetic testing or not [1-3], on whether to participate in surveillance programmes [4,5], or to accept prophylactic surgery [6-8]. It also influences levels of psychological distress [7,9,10].

Despite the fact that genetic counseling provides information regarding objective risks, there is frequently a contrast between the perception of the risk of developing a tumour and being a carrier of a genetic mutation and the objective risk [11,12]. These data imply that, apart from cognitive factors, the perception of risk is also influenced by various factors [13]. Literature evidenced that, age, together with other socio-demographic factors, as for example the education, the employment or the spirituality influenced moderately the risk perception. Some studies stated that younger women are more likely to perceive higher risk of developing breast cancer then older, while other studies concluded no significant relationship between age and perceived risk [14]. Only one study reported that employed women were more likely to overestimate their risk compared to their actual risk [15]. Findings on the relation between education and perceived risk concluded that women with high school or less education were more likely to be either unaware of their risk or overestimate their risk, whereas women with college education were less likely to have an optimistic bias [14]. The role of religion in health care decisions and perceived risk among people at increased genetic risk has not been deeply investigated yet. It exists a certain kind of religious fatalism (a belief that some issues are beyond human control but just in God's hands) that may influence the subjects' conceptions of how disease occurs and of how much they can be at risk for developing a particular disease based on family history [16,17]. On the basis of this kind of fatalism we may hypothesize that the perception of the risk is lower for subjects with high spirituality, as demonstrate by JM Quillin research [17]. Furthermore many studies focused on the role played by psychological distress levels and by the personal and family history of tumour in filtering, modifying and completing relevant information, concerning the objective risk, affecting in this way the risk perception of developing the disease[11,12,14,18].

As regards the relationship between the psychological distress and the risk perception findings are opposing. In fact several studies revealed a correlation between high distress levels and high risk perception, while few researches showed no correlation between these two variables [14,18].

As far as the family history of tumour is concerned, women with a personal and a family history of cancer usually perceived their risk of developing the disease as higher than that of other women. Nevertheless, comparing the risk perception with an objective estimation of the risk (Claus, Gail or BRCA-PRO models), the women affected by cancer and with a family history of tumour are more accurate in their risk estimation than women with a family history of tumour but healthy [11,12]. Women involved in several studies that revealed an overestimation of the risk perception are usually referred by an affected relative or by health care setting, while the studies that found an underestimation of the risk perception involved women referred by the community. The importance of risk perception in affecting the decisional making process of the counselee and the relationship between the risk perception and other psychological variables are key issues in the research on genetic counselling across different countries.

Nevertheless, in Italy, the risk perception has been little studied and counselors still miss relevant information like: how the risk perception is spread on Italian population, how the risk is associated to other psycho-social variables and if the risk perception is accurate or not compared to objective methods of risk estimate[19].

The main aim of this research is to study the risk perception among a population enlisted by a department of genetic counseling in the centre of Italy.

In particular, this study evaluates the perception levels of the risk to be a mutation carrier and to develop a related-cancer, the association between perceived risk and medical/demographic/psychological variables and the adequacy of the perceived risk compared to the objective risk estimated by BRCA-PRO model [20,21].

## Methods

### Participants

From February 2007 to November 2008, 153 subjects were submitted to genetic counseling at the Unit for Hereditary Breast and/or Ovarian Cancer of the "Regina Elena" National Cancer Institute of Rome. The analysis was carried out on a sample of 130 subjects who had cancer of the breast and/or ovaries and/or a family history of tumours (at least one first-degree affected relative). Twenty-one subjects did not complete the questionnaires and psychological tests, two were illiterate.

### Genetic counseling procedures

Subjects who requested counseling to the Unit for Hereditary Breast and/or Ovarian Tumours of "Regina Elena" National Cancer Institute of Rome were referred by their physician or came spontaneously under suggestion from relatives, friends, other oncologic patients or mass media information.

The counseling multidisciplinary team included an oncologist/counselor, psychologist, and geneticist. The counseling modalities were designed using a multistep approach as follows:

During the first visit the oncologist/counselor, supported by the psychologist, supplied the patient with information about hereditary cancer syndromes, the mutation of BRCA1/BRCA2 genes, and their involvement in the onset of cancer of the breast and/or ovaries. Further information is supplied regarding transmission, the possibility of prevention and treatment.

Afterwards, the physician asked the patient to sign in an informed consent to collect the family history in order to assess the genetic and cancer risk estimation.

Furthermore, risk estimation and eligibility or non eligibility status for genetic test was also performed following the Modena Criteria [[22,23], http://www.com.unimo.it/com2000/hbc/alberi/lineeg.htm].

Applying these criteria, subjects were classified as eligible if they were at "high risk", or non eligible if they resulted at "intermediate, or slightly increased risk", as described in Table 1. Only the eligible subjects were proposed to give a blood sample during a second counseling section after 14 days. This lapse of time was chosen to give subjects the time to elaborate the information and to decide with greater awareness.

**Table 1:** Modena Model

High risk			Pedigree Classification	
I) at least 3 relatives diagnosed with BC (or OC) in 2 different generations	II) one BC/OC case is a first degree relative of the other 2 (of the other 1 if the first criterion is not fulfilled)°	III) at lest one case has been diagnosed at the age ≤ 40 or with bilateral BC		
*	*	*	Hereditary	HBC/HBOC

*	*		Suspect Hereditary	SHBC/SHBOC

*		*	Suspect Hereditary	SHBC/SHBOC

BC diagnosed at age ≤ 35, regardless of family history			Early onset	EOBC

BC and OC in the same woman, regardless of family history			Breast and Ovarian Cancer	BOC

**Intermediate Risk**				

*			Familial	FBC/FBOC

	*	*	Strongly suspected Familial	SFBC/SFBOC

Male BC, regardless of family history			Male Breast Cancer	MBC

**Slightly Increased Risk**				

	*		Suspected Familial	SFBC/SFBOC

		*	Suspected Familial	SFBC/SFBOC

BC/OC without any of the described criteria			Sporadic Breast Cancer	SpBC/SpOC

BC - Breast Cancer; OC - Ovarian Cancer; H - Hereditary; F- Familial; S - Suspect; s - sporadic; M - Male

During the second visit the oncologist/counselor, supported by the psychologist, asked the patient his/her consent for the blood sample.

After six months the subjects knew the genetic test result. During this third visit the physician and the psychologist together communicated the outcome of the test, the possible involvement of the family into genetic counseling and the risk-reducing strategies, they help the subjects to express emotions, doubts, and requests focused on the genetic test outcome and on how to communicate the outcome to the sibling or children [24,25].

The local Ethic Committee approved the counseling procedures.

At the end of each counseling session the psychologist asked to the patients an informed consent to complete questionnaires and psychological tests.

During the second counseling step, only eligible subjects were proposed to give the blood sample; while for the others or non eligible subjects were organized an "ad hoc" surveillance programmes. This study refers to the data obtained by the questionnaires completed after the first genetic counseling session by 130 subjects. The sample was made up of eligible and non eligible subjects.

### Instruments

The questionnaires and psychological tests evaluate the following variables.

#### Demographic and medical characteristics

Data regarding age, geographic origin, civil status, number of children, education, religion and whether they were religious-practicing or non-practicing, eligibility, pathology, number of relatives affected by cancer of the breast and/or ovaries and the total number of relatives affected by any type of tumour were collected.

#### Cancer Risk Perception (CRP)

An item adapted from previous research was used to evaluate the perception of the risk of developing a tumour: "Indicate with a cross, on a scale from 0 to 100, that which you think is your current percentage risk of developing a tumour, or redeveloping a tumour of the breast and/or ovaries" [26,17].

The answer was given on a visual analogue scale from 0 to 100 (100 corresponds to the highest risk). The scale is a ten centimetres line and each millimetre corresponds to one point percent.

#### Genetic Risk Perception (GRP)

An item adapted from previous research was used to evaluate the perception of the risk of being a carrier of the genetic mutation BRCA1/BRCA2 [10]. "Indicate with a cross, on a scale from 0 to 100, which you think is your current percentage of being a carrier of the genetic mutation which causes cancer of the breast and/or ovaries." The answer was given on a visual analogue scale from 0 to 100% (100 corresponds to the highest risk). The scale is a ten centimetres line and each millimetre corresponds to one point percent.

#### Objective cancer and genetic risk assessment by BRCAPRO model

Data of the family pedigree were inserted (in a separate moment without the presence of consultant) into the computer programme "Cancer-Gene-Program" (that is based on the BRCAPRO evaluation model) to evaluate the risk of being a carrier of the BRCA1/BRCA2 mutation and the risk to develop breast and/or ovarian tumour[20,27,28].

This programme uses Mendelian genetics and the Bayes theory to estimate risk considering the following factors: the number of relatives affected and not affected by a tumour of the breast and/or the ovaries, age at onset, number of generations affected, tumour of the breast in men. The final estimation results are two percent values, one for the risk of being a carrier mutation and one for the risk to develop a tumour. This model has been used on large samples and in many countries. It considers factors which other models omit, and its validity and sensibility (by identifying subjects with probable genetic mutation) has been demonstrated in six centres of genetic consulting [19,29,30]. This software is easily available via the internet and it is also user-friendly. The last version is CaGene5, available from the official web site: http://www8.utsouthwestern.edu/utsw/cda/dept47834/files/67943.html.

#### Accuracy of the perception of risk

The percentage risk of developing a tumour and of being a carrier of a genetic mutation evaluated by BRCAPRO were compared to the percentage of perceived risk in order to assess the adequacy of the perceived risk compared to the objective risk (more details in the statistical methods section).

#### Anxiety and Depression

The Hospital Anxiety and Depression Scale (HADS) [31], Italian version [32] is used in literature to evaluate the psychological distress in a non-psychiatric setting. It is composed of two scales of 14 items, 7 regarding anxiety and 7 regarding depression. The two scores can be calculated separately with three cut-offs: normal anxiety and depression (0-7), borderline anxiety and depression (8-10), disturbance due to anxiety and depression (≥11). By calculating the sum of the two scales, it is possible to identify the presence of disturbance in adaptation(cut-off 13-18), or an episode of heavy depression (cut-off ≥ 19). No psychological distress is evidenced if the sum of the two scores totals <13.

All instruments used were chosen on the basis of the following characteristics: validation, internal reliability and previous use in literature for populations comparable to the one from which the sample for the present study was drawn.

### Statistical methods

Descriptive statistics were used to summarize pertinent study information.

The association between variables was tested by the Pearson Chi-Square test.

A paired sample t-test was used to compare the mean values of the subjective perception of risk, with the objective risk, estimated by BRCAPRO.

The percentage risk of developing a tumour and of being a carrier of a genetic mutation evaluated by BRCAPRO were compared to the percentage of perceived risk in order to assess the adequacy of the perceived risk compared to the objective risk. To make this comparison, Bluman et al. in 1999 [33] calculated the quartiles (≤ 25%, 26%-50%, 51%-75%, ≥ 76%) of both the percentage values of objective and subjective risk and after that they make a comparison between the two values. The variable, resulting from this comparison, categorizes the subjects in overestimators, accurate estimators and underestimators.

Differences between groups ("corrected", "under" and "over" estimators) with Kruskal-Wallis non parametric test were analyzed for age, number of relatives affected by cancer and for distress levels.

Concordance between the subjective perception of risk and the objective risk estimated by BRCAPRO was assessed using Cohen's k coefficient of agreement [34]. Landis and Koch proposed categories for judging K values: K less than 0.0 was considered poor, 0.00 to 0,20 was light, 0.21 to 0.40 was fair, 0.41 to 0.60 was moderate, 0.61 to 0.80 was substantial and 0.81 to 1.00 was perfect [35]. Given ratings on a K-level categorical variable, the marginal homogeneity test was used for calculated agreement between two rates summarized by a K × K cross-classification table.

Given the small numbers, statistical analyses cannot be performed to assess the differences between male and female in risk perception. The SPSS (11.0) statistical program was used for the analyses.

## Results

### Description of the sample

The average characteristics of the sample of 130 subjects (women/men = 119/11) are reported in Table 2 and 3.

**Table 2:** Descriptive results

N = 130 subjects		
**Women/Men = 119/11**		

	**Median**	**Range**

Age	47	19-77

Number of relatives affected by tumours of the breast and/or ovaries	2	0-6

Number of relatives affected by other types of tumour	4.5	0-18

	**Frequency**	**%**

**Geographical Area of Origin**		

Central Italy	100	77

Other areas (South-North-Abroad)	30	23

**Civil Status**		

Single	58	44.6

Married	72	55.4

**Number of children**		

No children	43	33.1

1 child	26	20

+ children	61	46.9

**Education**		

Primary (age 5 to 14)	27	20.8

High school (age 14 to 19)	65	50

University	38	29.2

**Profession**		

Worker	87	66.9

Unemployed	43	33.1

**Eligibility**		

Eligible	81	62.3

Non-eligible	49	37.7

**Pathology**		

Affected	42	32.3

Non-affected	88	67.7

**Table 3:** Descriptive results

	*Mean*	*Range*
Anxiety	7.9	0-16

Depression	5.1	0-15

Cancer Risk Perception*	38.9	0-100

Genetic Risk Perception**	39.9	0-86.8

BRCA pro Cancer Risk	10.6	0-99.1

BRCA pro Genetic Risk	18.7	0.10-66.5

	***Frequency***	***%***

***Adequacy of the cancer risk perception***		

Overestimation	65	56.9

Adequate Estimation	38	31.9

Underestimation	13	11.2

***Adequacy of the genetic risk perception***		

Overestimation	77	66.9

Adequate Estimation	30	26.1

Underestimation	8	6.9

*14 subjects were unable to report their risk levels for cancer of the breast and/or ovaries

**15 subjects were unable to report their level of risk of being a carrier of the genetic mutation of the BRCA1 and BRCA2 genes

#### Subjective and objective risk

The mean percentage regarding the subjective risk of developing a tumour and of being a carrier of the genetic mutation were 39% and 40%, respectively.

The mean percentage regarding the objective risk, calculated using the BRCAPRO model, of developing a tumour and of being a carrier of the genetic mutation were 11% and 19%, respectively.

#### Anxiety and Depression

The total mean score was 13, with 24% of the subjects suffering one episode of major depression and 19% experiencing the presence of some disturbance in adaptation.

A mean score of 8 was found for the single scales (borderline anxiety) and of 5 (normal depression). A total of 25% had borderline anxiety levels and the same value was found in subjects suffering from anxiety.

Depression was found in 9% of the subjects, while 15% were borderline.

### Association between medico-demographic variables and risk perception (table 4 and 5)

**Table 4:** Associations between the perception of risk (CRP-GRP) and Medical-Demographic variables

	N	Mean	Std. Deviation	P (2-tailed)
**ELIGIBILITY**				

**Cancer Risk Perception**				
Non-Eligible	44	32.82	21.87	
Eligible	72	43.04	24.13	.024*

**Genetic Risk Perception**				
Non-Eligible	43	29.11	21.92	
Eligible	72	46.45	21.96	.000*

**PATHOLOGY**				

**Cancer Risk Perception**				
Non-Affected	84	38.63	21.14	
Affected	32	40.89	30.35	.712

**Genetic Risk Perception**				
Non-Affected	83	37.90	22.99	
Affected	32	45.23	23.74	.108

**Table 5:** Associations between the perception of risk (CRP-GRP) and Medical-Demographic and Psychological variables

	Cancer risk perception	Genetic risk perception
**Anxiety**		
Pearson coefficient	0.050	0.087
P (2-tailed)	0.596	0.355

**Depression**		
Pearson coefficient	-.031	.072
P (2-tailed)	.742	.537

**Age**		
Pearson coefficient	-.068	-.030
P (2-tailed)	.468	.747

**Number of relatives affected by breast and/or ovarian cancer**		
Pearson coefficient	.053	-.082
P (2-tailed)	.569	.386

**Number of relatives affected by other types of tumour**		
Pearson coefficient	-.149	-.139
P (2-tailed)	.111	.140

**BRCA pro Cancer Risk**		
Pearson coefficient	.254	
P (2-tailed)	.006	---

**BRCA pro Genetic Risk**		
Pearson coefficient		.322
P (2-tailed)	---	.000

Of all the medical-demographical variables, only the condition of eligibility was found to be statistically associated to the perception of risk (Table 4).

The subjects who were eligible for genetic testing had a significantly higher perception of risk compared to the non-eligible people (CRP = 43%vs33%, p = 0.024; GRP = 46%vs29%, p < 0.000).

Despite the fact that the pathology was not found to be associated with the perception of risk, the affected subjects were found to have higher percentages than healthy subjects (CRP = 41%vs39%, P = 0.712; GRP = 45%vs38%, P = 0.108).

### Associations between psychological distress and risk perception (table 5)

No correlation was found between the levels of perception of risk and anxiety (CRP r = 0.050 p = 0.60;GRP r = 0.087 p = 0.35) and depression (CRP r = -0.31 p = 0.74;GRP r = 0.072 p = 0.53).

No correlation was discovered between distress levels and family history of tumour (r = 0.050 p = 0.60).

No significant differences in distress levels were revealed between affected and non-affected subjects (Distress = 12.42 vs 13.32 p = 0.46) and between eligible and non-eligible subjects (Distress = 18.82 vs 13.37). However, the non-affected and the non-eligible subjects were above the cut-off point of disturbance in adaptation.

### Associations between objective and subjective risks (table 5)

The subjective risk was found to be correlated to objective risk BRCApro (CRP r = 0.254 p = 0.006; GRP r = 0.322 p < 0.000).

However, the percentage levels of subjective risk were found to be significantly higher than for objective risk (CRP = 39%vs11%, p < 0.000; GRP = 40%vs19%, p < 0.000).

### Accuracy of the perception of risk (table 3)

Compared to the objective risk a significant percentage of individuals overestimated the risk of developing a tumour (57%, p < 0.000), while only 11% underestimated the risk. The remaining 32% made an accurate estimation. Concerning the risk of being a carrier of the genetic mutation a significant number of subjects overestimated the risk (67%, p < 0.000), while 7% underestimated it and 26% had an accurate perception.

Eligible subjects made a significantly more accurate estimate of their risk compared to non-eligible ones, CRP(P = 0.001) and GRP (P = 0.006).

## Discussion

The subjects with less cancer affected relatives significantly overestimated their risk of being mutation carrier (p = 0.028). No association was found between other medical-demographic or psychological variables and the accuracy of the risk estimate.

The results show that most of the sample overestimated their cancer and genetic risk. This Italian sample, under this aspect, does not differ from samples of subjects with higher risk of breast cancer and/or ovary tumours from other countries, like Spain [17], United Kingdom [36], USA [10], Netherlands [7] and Australia [37].

The relevant overestimation of risk leads to the belief that information gathered during counseling sessions does not adequately reach the patient, as elsewhere reported in literature [5,38,39].

However, in the present study this misunderstanding seems to be associated to eligibility conditions and to the number of cancer affected relatives. Indeed, the subjects with a high family history of tumour (usually the eligible subjects for genetic testing) seem to underestimate or make an accurate estimation of their risk, while subjects with at least one affected relative, but not eligible (Intermediate or Slightly increased risk), had a percentage of overestimate of risk significantly higher. Our hypothesis is that the subjects eligible for a genetic test, having a high number of relatives affected by tumours and often stricken themselves, are not only more open to information regarding their risk, but also more aware in comparison to subjects with familiarity or with sporadic events of breast and/or ovarian tumours in their family [10,14,40].

As far as the association between psychological variables and risk perception is concerned, some studies evidenced that there is a positive correlation between the perception of risk and levels of psychological distress. However, in this study, no such correlation was found, despite the fact that the psychological distress levels reached the cut-off value of disturbance in adaptation. We do not have an Italian regulatory sample of reference for HADs which considers not only subjects with tumours but also healthy subjects. However, in a population of women with breast cancer the percentage of subjects unable to adapt to the situation was of 24% (19% in our sample) and of 9,8% with at least an episode of major depression (24% in our sample) [32]. These two scores, as set forth in the methods, are obtained adding the score of each individual measure of anxiety and depression. Taking this into consideration, it is interesting to note that in our sample the raising of the percentage of the subjects with at least one episode of major depression, with respect to regulatory samples (24% vs 9.8%), derives from the elevation of the anxiety scale: 25% of borderline anxiety samples and 25% with anxiety disorders. Despite the fact that a high psychological distress is shown, mainly consisting of an element of anxiety, there is no association between the risk perception "per se" and anxiety or depression levels and neither between the accuracy of risk perception and anxiety or depression levels.

This could depend on the fact that the HAD's scale, although largely used in genetic counseling for hereditary tumours, reveal a type of "general" psychological distress linked to a pathological event rather than a "cancer-specific" distress.

Punctual correlations between distress and perception levels found in literature has been evidenced through the use of cancer-specific instruments (for measuring distress levels due to cancer worries) such as the Cancer Worry Scale of Lerman, or the Impact of Event Scale of Horowitz [36,41]. The latter can be adapted for a kind of distress due to specific pathologies. Unfortunately, these tests are not still validate in all country - specific languages, (i.e. Lerman test is actually under validation in Italian Language through a multi-cohort research project coordinated by the National Committee PSICONCOGEN of Italian Society of Psycho-Oncology) and the experiences about the use of cancer - specific questionnaires in genetic counseling in Italy doesn't exist yet.

As set forth in the introduction section we suppose that the spirituality has a negative correlation with the risk perception. No difference has arisen between religious and non-religious subjects; however, one have to consider as a limit the measure of religion and religiosity which is not overtly articulated and thorough as far as prayers and the degree of emotional and cognitive involvement in these rites are concerned.

### Limitations

Limitations to the current study should be noted. To begin, it is important to take into consideration the self-selection bias. The general overestimation of the risk can be due, from one part to the self-referral way of inclusion in the study and to the other part, to the fact that all the eligible subjects for this study had almost one first degree relative affected by cancer of the breast or ovaries. In actual fact, the subjects of this study asked for a visit because they thought their chances of having a mutation and/or their breast cancer risk was high. Secondly, the BRCAPRO evaluation model can introduce some limitation (that is an underestimation of the risk), not considering in the calculation of the risk relatives with less than first degree of kinship. Moreover, the instrument used to measure the perceived risk, the numerical visual analogue scale, sometimes lead the patients to overestimate their own risk [13].

Thirdly, it could be difficult to know how generalizable these results from a select sample of subjects coming from the centre of Italy are to populations that come from other parts of Italy or to other ethnic groups.

## Conclusions

In Italy, where health care is mainly a public service concern, and cancer genetic counseling is a relatively new concept and is almost invariably offered within the framework of clinical research units, the variable "perception of risk" has been very little investigated [18].

The present study attempts to describe the perception of risk in subjects who have requested oncological genetic counseling in a sample of Central Italy. The results are similar to other studies carried out in other countries in the following ways: general overestimation of the risk, inaccurate perception compared to systems of objective calculation and an underestimation or more accurate estimation in those subjects with eligibility criteria.

### Practice Implications

From information derived from this study we find that the doctors working in the oncological genetic counseling in Italy, as well in other countries, are face an exacting task to impart information to people who often have high anxiety levels (they do not usually reach pathological limits) and an exaggerated perception of personal risk of having a genetic mutation and/or a tumour. In particular we found that the misperception of the risk is higher for the subjects with familiarity or with sporadic events of breast and/or ovarian tumours in their family (at intermediate or slightly increased risk, Table 1). For that reason it appears necessary that more attention, from a cognitive and emotional point of view, must be paid both by the physicians and the psychologist especially for these target of subjects (at intermediate or slightly increased risk, Table 1). It might be important that physicians verify, step by step, the level of consultants understanding, asking consultants opinions and facilitating answers or doubts regarding the familial risk information. Psychologist, might facilitate this communication between consultant and physician. Moreover, during the psychlogical talk, it might also facilitate the awareness process which necessary involves cognitive and emotional aspects concerning the cancer and genetic risk information.

Reported data were collected after the first genetic counseling session and cannot therefore be subsequently checked. It is our intention to await until data relative to psychological follow-up after counseling are completed that is to say 48 months after the outcome of genetic test with the aim to evaluate the evolution of the psychological impact of genetic counseling as well as to assess the possibility of new or improved interventions.

## Competing interests

The authors declare that there are no financial or non-financial competing interests (political, personal, religious, ideological, academic, intellectual, commercial or any other) in relation to this manuscript.

## Authors' contributions

AC main author project of the study and interpretation of the data, CV and BM patient's data collection, data analysis and interpretation of the data, FMS, FC and AS project of the study and study coordinator.
